# The Inferto-Sex Syndrome (ISS): sexual dysfunction in fertility care setting and assisted reproduction

**DOI:** 10.1007/s40618-021-01581-w

**Published:** 2021-05-06

**Authors:** G. Luca, S. Parrettini, A. Sansone, R. Calafiore, E. A. Jannini

**Affiliations:** 1 Unit of Andrology and Endocrinology of Reproduction, Department of Experimental Medicine, University Medical School, 06129 Perugia, Italy; 2 Section of Endocrinology and Metabolism, Department of Medicine, Perugia, University Medical School, 06129 Perugia, Italy; 3 Chair of Endocrinology and Medical Sexology (ENDOSEX), Department of Systems Medicine, University of Rome Tor Vergata, Via Montpellier, 1, E Tower South. Floor 4, Room E413, 00133 Rome, Italy

**Keywords:** Erectile dysfunction, Infertility, Assisted reproduction, Sexuality

## Abstract

**Purpose:**

Infertility represents a peculiar social burden affecting more than 15% of couples, provoking it a real threat to the general quality of life and to the sexual health. The medicalization (diagnosis, therapy and follow up) of the lack of fertility is frequently a challenge in term of personal and couple’s involvement. In particular, while the *Assisted Reproductive Technology* (ART) has allowed many infertile couples to achieve pregnancy, the therapeutic process faced by the couple bears a strong psychological stress that can affect the couple's quality of life, relationship and sexuality. Despite infertility affects both female and male sexual health, only recently the interest in the effects of ART on the couple's sexuality has grown, especially for women.

**Methods:**

A literature research on the sexual dysfunction in fertility care and particularly in ART setting was performed.

**Results:**

Literature largely found that intimacy and sexuality appear specifically impaired by intrusiveness of treatments and medical prescriptions. Moreover, there is a close relationship between emotional, psychological and sexual aspects, which can be integrated in the new concept of Inferto-Sex Syndrome (ISS) that can impair the ART treatment outcomes. Evidence demonstrates that the assessment of sexual function is necessary in couples undergoing diagnosis of infertility and ART.

**Conclusion:**

A close relationship between infertility and sexuality, both in the female and male partners, was detected. ART treatments may heavily impact on the couple's psychosexual health. A couple-centred program for the integrated management of psychological and sexual dysfunction should be considered in the context of ART programs.

## Introduction

Since the first live birth resulting from in vitro fertilization (IVF) in 1978, a growing number of pregnancies derived from assisted reproductive techniques (ART) has been observed, with an impressive growing trend in the last 15 years. The use of ART has allowed many infertile couples to achieve pregnancy, but the therapeutic process is a strong psychological stress condition that can affect their quality of life as well as the couple's relationship and sexuality.

The diagnosis of infertility is by itself a powerful stress factor, mainly for women who feel impaired their female identity, often burdened with social pressure, but also for men who live with discomfort the inability to procreate, especially if the infertility is caused by a male factor [1–3]. Considering that up to 83% of infertile couples report feeling a social pressure to conceive [4], the decision to access ART increases distress of the couple, also burdened with the costs, the ethical implications and the difficulties in managing eventual failures [1]. This stressful condition has been associated with severe anxiety and depression in both partners, with possible immunological alterations and a lower likelihood of clinical pregnancy in in vitro fertilization (IVF) procedures [5].

Infertility affects both female and male sexual health, but only recently the interest in the effect of ART on the couple's sexuality has come into a sharper focus. A recent Italian survey showed that in the Infertile Care Units the attention on sexuality was rather limited, with a strong discrepancy between the two members of the couple, with greater attention paid to male sexuality [6].

**Figure 1.**
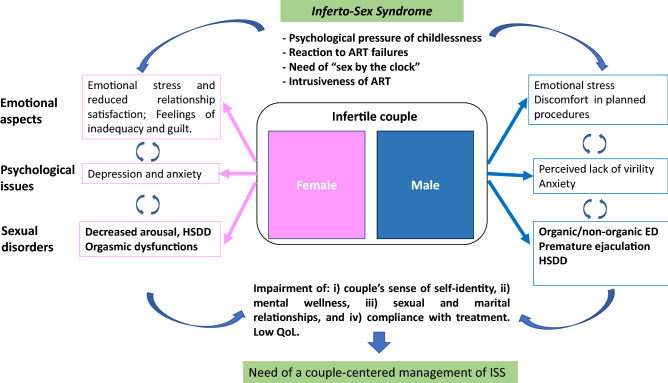
Infertility makes a real challenge to the sexual life and SD are an emerging paradigm as a typical “couple disorder”, especially in unique psychosocial set of ART treatment for infertility. A couple-centred approach in the infertility treatment plan is mandatory, grounded on a multidisciplinary team for the comprehensive management of ISS, as all the emotional, relational and, inevitably, sexual aspects of infertile couples. *ART* assisted reproductive technology. *ED* erectile dysfunction. *HSDD* hypoactive sexual desire disorder. *ISS* Inferto-Sex Syndrome

The close relationship between infertility and sexuality, both in the female and male, and the need to contextualize sexuality within the infertile couple undergoing ART treatment, may be framed in a broader dysfunctional dimension, conceptualized in the Inferto-Sex Syndrome (ISS) (Fig. 1). The utility to introduce a new taxonomic term is not merely conceptual, but carries the message to consider sexual health along with the reproductive one. Too frequently, sexuality and fertility have been and are considered two different and separate fields, the former studied by the psychosexologists and the sexual physicians, the latter by the gynaecologists. The ISS aims to stress the need of an integrated and shared diagnosis and therapy, considering how sexuality and fertility are, in our species, imbricated, overlapping and strictly correlated functions.

Finally, because of the effects of ART treatments on the couple's psychosexual health, a couple-centred program for integrated management of psychological and sexual dysfunction in the context of ART should be assessed.

## Infertility and sexual function

Infertility is defined as inability of a couple to conceive naturally after 1 year of regular unprotected sexual intercourses [7]. At least 50 million couples worldwide experience infertility, a social burden that affect more than 15% of couples [8]. Infertility involves both partners of a couple, therefore, it has different effects on psychology and sexuality in men and women, assuming gender differences in the personal experience of the childlessness. As infertility is always a disease of the couple and the couple is to be treated [9], a similar approach must be pursued for the assessment of the associated sexual dysfunctions (SD). The ISS definition is based on this assumption, as an integrated set of SD in both partners of couples seeking fertility, which must be evaluated and managed with an integrated approach aiming to protect the psychosexual health of the couple and improve the outcomes of ART.

Up to the present date, the difficulty in framing the real dimension of the psychological and sexual impact of infertility is due to, first of all, different cultural habits. In fact, many traditional backgrounds, religions and societies still consider infertility a stigma. Culture, considered as an organized system of beliefs and values, determines all human behaviors including the sexual ones: social models play an important role in determining conducts about parenthood, fertility and sexuality [10]. The fragile balance between what the individual needs and what the society finds to be normal can exert a deep impact on well-being and sexual behavior [11]*.* Thus, the cultural differences can impact the sex drive in an infertility context [12]. A substantial culture-dependent influence has been reported in each partners’ perception of SD, in their views on sexuality and their own levels of sexual functioning [13, 14]. In addition, ART can collide with sociocultural and religious practices, separating the sexual act from the reproductive function that is the dominant purpose in some cultures. Consequently, the results of SD prevalence in male and female infertility are extremely variable and difficult to compare across studies.

Therefore, surveys on sexuality are based on standardized clinical interview and questionnaires that vary in different studies and that are not always validated, producing many biases due to self-assessment by the partners [15] and being sometimes hard to compare.

Finally, SDs can appear in both partners of the infertile couple in every step of the diagnostic-treatment protocols and might provoke problems in every stage of sexual response, i.e. from desire to arousal and to orgasm.

### Female sexual dysfunctions and infertility

Female sexual dysfunction (FSD) is a complex and multidimensional disorder that has a wide spectrum of symptoms and severity, including non-organic and organic risk factors, such as endocrine diseases [16], cardiovascular diseases [17] and health-risk behaviors and lifestyles [18, 19]. Infertility might also be part of the clinical phenotype of some of these conditions [20], such as polycystic ovary syndrome [21], or a consequence of the necessary treatments. The effects of infertility on female sexual well-being have only recently elicited an increased interest, this delay reflecting how female sexuality is underdiagnosed and poorly studied, compared to its male counterpart. Several tools have been developed and validated in the last decades to provide a reliable assessment of female sexual health, among which the Female Sexual Function Index (FSFI) score, a 19-items multidimensional scale for assessing different phases of the female sexual cycle (desire, arousal, orgasm, sexual satisfaction and dyspareunia), is the most commonly used instrument in both the clinical and research settings, both in its long [22] and abridged form [23].

Following a diagnosis of infertility, a woman wishing to conceive may face obsessive thoughts, anguish, perception of inadequacy of her social role, guilt for her own fertility; in case of infertility of the partner, perceived as offense towards her, a tendency to social self-isolation, and sometimes envy of other women's pregnancy have been observed [24–26]. As a result, sexuality is heavily affected, thus women usually report major sexual issues such as decreased arousal, hypoactive sexual desire disorder (HSDD) or orgasmic dysfunctions [15] (Table 1).

**Table 1:** Studies of sexual dysfunctions in infertile female population

Study	Study design and population	Infertile women (case group)	Fertile women (control group)	FSD assessment	Psychological assessment	QoL assessment	Infertility duration	Type of ART treatment	FSD prevalence and results
Nelson et al. 2008 [27]	Cross-sectional survey	121	N.A	FSFI	CES-D	SF-36	23.5 ± 24 months	N.A	26% of women had FSFI score <26.55. FSFI modestly correlated with male IIEF score. Negative correlation between FSFI and CES-D scores.
Oskay et al. 2010 [41]	Case control study	308	308	FSFI	N.A	N.A	6.0 ± 4.45	N.A	61.7% of the infertile women scored FSFI < 26.55. FSFI in all domains (except pain) in infertile women was adversely affected by duration of infertility and duration of treatment
Millheiser et al. 2010 [40]	Case control study	119	99	FSFI + Not validated questionnaire about sexual satisfaction	N.A	N.A	N.A	IUI, IVF, or ovulation induction	40% of infertile women vs 26% in controls reported SD. Infertile women had significantly lower scores in the desire and arousal domains and lower frequency of intercourse.
Iris et al. 2013 [36]	Case control study on relation between FSD and **infertility duration**	174 women with primary infertility	635	FSFI	N.A	N.A	3 groups (I = < 2 year; II = 2–5 years; III = > 5 years)	N.A	No differences between fertile and infertile women. Prolonged infertility duration negatively affected female sexual life (lower FSFI scores).
Furukawa et al. 2012 [34]	Case control study on dyspareunia and FSD	75	210	FSFI	PHQ-9	N.A	N.A	N.A	The rate of dyspareunia and sexual dysfunction were similar between infertile and control groups. Secondary infertile women were at higher risk.
Pakpour et al. 2012 [29]	Cross-sectional study	604	N.A	IV-FSFI	HADS	SF-36	N.A	N.A	56% of infertile women had FSD. Depression was a strong predictor of sexual problems.
Hentschel et al. 2008 [32]	Cross-sectional survey	96	119 women looking for surgical sterilization	FSFI	N.A	N.A	N.A	Women not yet in ART	No differences between infertile women and those undergoing tubal ligation.
Carter et al. 2011 [113]	Cross-sectional cohort study	50	N.A	FSFI	CES-D IES ADAS RCS	MOS SF MCS MOS SF PCS	N.A	Oocyte donation	46.8% of women looking for oocyte donation experienced FSD. Infertility negatively impact QoL and emotional well-being.
Davari Tanha et al. 2014 [39]	Cross-sectional study aiming to evaluate sexual function according to the type of infertility	191 primary infertility and 129 secondary infertility	87	FSFI	N.A	N.A	N.A	N.A	SDs were higher in infertile women. Women with secondary infertility suffer more from impaired sexual function compared with those with primary infertility.
Bakhtiari et al. 2015 [101]	Cross- sectional study	236	N.A	DSM questionnaire for sexual dysfunction	N.A	N.A	N.A	N.A	55.5% prevalence of FSD in women undergoing fertility treatment. Age, sexual satisfaction, female infertility cause and history of mental illness had a significant effect on the probability of experiencing SDs.
Suna et al. 2015 [28]	Cross-sectional study	142 (3 groups related to cause of infertility: A = female factors; B = male factors; C = unknown factor	N.A	FSFI	BDI	N.A	The mean duration of attempted conception for groups A, B and C was 3.7 ± 3.7, 4.8 ± 4.2 and 3.3 ± 3.2 years, respectively	N.A	No statistically significant difference in the FSD prevalence and in BDI scores among the three groups. Infertile females with SDs were more likely to have depressive symptoms.
Czyżkowska et al. 2014 [45]	Cross—sectional study on sexual satisfaction in infertile women	50	50	SSS Scale Mell-Krat Scale	BDI	FAM-III	Classified in three groups (42% 1–2 years, 40% 3–4 years, 18% > 5 years)	All women treated for infertility, no distinction on type of treatment	90% in infertile vs 26% of fertile women reported Mell-Krat Scale indicative of SDs. Infertile women reported lower sexual satisfaction and more maladaptive patterns of dyadic functioning.
Ozkan et al. 2016 [26]	Prospective study	56 women with infertile male partners	48	FSFI	BDI STAI MAS	N.A	N.A	First IVF cycle	Similar prevalence of SDs between the two groups. Women with infertile partners experienced sexual problems related to lubrication and pain and higher level of depression.
Lo et al. 2016 [30]	Cross-sectional survey	159	N.A	FSFI	N.A	FertiQoL	22 months (mean)	N.A	The incidence of FSD (desire, arousal, lubrication and orgasmic disorders and pain) was 32.5%, 15.7%, 19.3%, 22.3%, 33.1% and 15.1%, respectively. Infertile women with SDs had significantly worse QoL.
Zare et al. 2016 [37]	Cross-sectional study	110	110	GRISS-F1	N.A	N.A	4.85 ± 3.53 yrs	N.A	No significant difference between fertile and infertile women in terms of sexual problems (15.5% vs 15.4%). Most SDs in infertile women were non-communication, while in fertile women were infrequency.
Alihocagil Emec et al. 2017 [35]	Comparative study	137	142	FSFI	N.A	N.A	Classified in four groups, not mean available	Hormone therapy, vaccination, IVF	76.8% of the women without infertility problems and 78.8% of infertile women had FSD (p > 0.05).
Diamond et al. 2017 [44]	Cross section analysis aiming to determine the characteristics of SDs in women with PCOS *vs* unexplained infertility (UI)	750 women with PCOS and 900 with unexplained infertility (UI)	N.A	FSFI FSDS	N.A	MD-PHQ SF-36 FertiQoL	41.8 ± 38.0 in PCOS group vs 34.3 ± 24.6 in IU group (Mean 37.7 ± 31.6)	N.A	No differences in sexual function between infertile women with polycystic ovary syndrome and those with unexplained infertility, despite phenotypic and biochemical differences in androgenic manifestations.
Barut et al. 2018 [91]	Case control	88 (42 with hypogonadotropic hypogonadism-HH)	N.A	FSFI	BDI	N.A	N.A	IVF	In the HH group, 64.28% reported SD vs 30.34% in the control group (infertile women without HH).
Shaharaki et al. 2018 [43]	Case–control study	164 (78 primary *vs* 71 secondary infertility)	115	FSFI	BDI	SQOL-F	N.A	Candidate for IVF	No significant differences between fertile and infertile women. Primary infertility was associated with lower FSFI and SQO-F scores and higher BDI score.
Ozturk et al. 2019 [42]	Cross-sectional and comparative analysis	96	96	FSFI	BDI	N.A	N.A	History of infertility treatment for at least 6 months	The SD rate in infertile women was higher than among fertile controls. (87.5% vs 69.8%).
Salomăo et al. 2018 [31]	Case–control study	140	140	FSFI	HADS	N.A	N.A	N.A	Infertile women had no increased risk of SD with respect to controls. Anxiety and depression increased the risk of SD.
Facchin et al. 2019 [105]	Observational study examining the association between infertility-related distress and FSD. The data were collected on the day of oocytes retrieval	269	N.A	FSFI FSDS-R NRS for dyspareunia	FPI	N.A	5.8 ± 3.7 years (not different between SD and no-SD groups)	IVF/ICSI	Women with higher infertility-related distress were more likely to report SDs.
Smith et al. 2015 [103]	Cross-sectional survey	136	N.A	SFQ	N.A	FertiQOL	Classified in four groups, not mean available	IVF	Women undergoing IVF scored significantly lower in sexual interest, desire, orgasm, satisfaction, sexual activity, and overall sexual function. Sexual problems predicted FertiQoL scores.
Karli et al. 2019 [104]	Prospective study of infertile patients with poor ovarian reserve *vs* unexplained infertility	146 (48 with poor ovarian reserve and 98 with unexplained infertility)	N.A	FSFI	N.A	N.A	3.7 ± 3.1 yrs in unexplained infertility vs 4.3 ± 3.6 yrs in poor ovarian reserve infertility	IVF (90.9% no previous IVF treatment)	FSFI score < 26.55 in 93.9% of patients with unexplained infertility and in 89.6% in patients with poor ovarian reserve. No significant difference between the two groups in each domain of desire, arousal, lubrication, orgasm, satisfaction and pain.
De Souza et al. 2018 [108]	Observed prospective study on vaginismus	425 IVF/ICSI cycles and 226 embryo transfers	N.A	Not validated questionnaire tailored to detect clinical trouble with TVUS (vaginismus)	N.A	N.A	N.A	IVF/ICSI	Seven cases of vaginismus were described.
Purcell-Levesque et al. 2018 [75]	Cross-sectional study	88 women seeking fertility treatment	N.A	Arizona Sexual Experiences Scale Global Measure of Sexual Satisfaction	Experiences in Close Relationships scale	N.A	4.49 ± 3.36 yrs	N.A	FSD varied from 14.8% to 58.0%. Desire and arousal were the domains in which more problems were reported Avoidance predicted low sexual satisfaction and pain.
Oindi et al. 2019 [33]	Analytical cross-sectional study	93	93	FSFI-Q	N.A	N.A	N.A	N.A	The FSD prevalence was 31.2% in the sub-fertile group and 22.6% in fertile control group. Subfertility type was not associated with SDs.
Gungor et al. 2019 [102]	Case–control	134	134	FSFI	BDI	SF-36	19.3 ± 4.6 months	IUI	Total FSFI score showed a lower sexual function for patients going to IUI (in particular sexual desire, arousal and satisfaction).

*QoL* quality of life, *SD* sexual dysfunction, *CES-D* Center for Epidemiological Studies Depression Scale, *FSFI* Female Sexual Function Index, *BISF* Brief Index of Sexual Functioning, *SFQ* Sexual Function Questionnaire, *FPI* Fertility Problems Inventory, *GRISS* Glombok-Rust Sexual Status Questionnaire, *SSS* Sexual Satisfaction Scale, *SF-36* Short Form 36, *FAM-III* Family Assessment Measure, *STAI* State-Trait Anxiety Inventory, *MAS* Marital Adjustment Scale, *MD-PHQ* Medical Outcomes Survey, *BDI* Back Depression Inventory, *FPI* Fertility Problem Inventory, *HADS* Hospital Anxiety and Depression Scale, *FertiQol* Fertility quality of life tool, *PHQ-9* Patient Health Questionnaire-9), *IUI* intrauterine insemination, *IVF* in vitro fertilization, *ICSI* intracytoplasmic sperm injection

In cross-sectional studies, a close relationship between sexuality and psychological wellness was evident in infertile female population. In fact, the prevalence of depression was significantly higher in women with SDs (up to 54% in some studies) [27, 28] and depressive symptoms have shown to be a predictor of SD [27–29]. The emotional and sexual effects translate into a deterioration of the quality of life [27, 28], especially with respect to relational aspects [30].

Several factors in the diagnostic and therapeutic protocol of infertility can negatively interfere with the emotional aspect, thus justifying a greater impact on the dimension of arousal, desire and satisfaction in female sexuality [27], more related to the relational dimension, and hence including a psychopathological dimension.

With respect to fertile female populations, case–control studies showed conflicting results. Some studies found no differences in the prevalence of FSD in infertile women, as compared to fertile controls [31–34]. In two studies conducted on a Turkish population, despite no differences in FSD prevalence between infertile and fertile women, the prevalence of FSD was very high [35, 36]. Probably, some cultural dimensions, such as the lack of formal sex education and the perception of sexuality as a taboo, contribute to the poor definition of the size of the problem [35]*. Zare *et al. found that the most impaired sexual dimension in infertile women was non-communication, i.e. the couple’s inability to talk about their sexual problem, while in fertile women the reduced frequency of sexual intercourse prevails [37].

In contrast, several studies showed a higher prevalence of FSD in infertile women. Indeed, the psychological burden of infertility—with the associated feelings of inadequacy, anxiety and depression [38]—might be a major contributor to the onset of FSD. Some studies have considered a population of women belonging to an infertile group or undergoing treatment for infertility, regardless of the diagnosis or treatment phase in which they were [39–44]. The prevalence of FSD assessed by FSFI score ranged from 40% [40] to 87.5% [42], always significantly higher than in the fertile women population used as control, being the arousal and libido domains of sexuality mostly impacted [40, 41].

It has been found that up to 90% of infertile women had a reduction of sexual functioning as compared to 26% of the fertile control population, scoring lower in terms of sexual satisfaction and significantly higher (maladaptive) on all six domains of dyadic functioning (task accomplishment, role performance, communication, affective expression, affective involvement). The authors speculated that infertility could be considered as a specific ‘crisis’, in which the quality of sexual function is closely associated with treatment procedures [45].

### Male sexual dysfunctions and infertility

Many studies have focused exclusively on the impact of infertility on male sexuality, with a prevalence of SD varying from 8 to 85% (Table 2). Similarly to what has been reported in women, the psychological burden of infertility is relevant for men as well, most commonly being associated with increased rates of depression [46].

**Table 2:** Studies of sexual dysfunctions in infertile male population

Study	Study design and population	Infertile men (case group)	Fertile men (control group)	SD assessment	Psychological assessment	QoL assessment	Infertility duration	Type of ART treatment	SD prevalence and results
Coward et al. 2019 [57]	Secondary analysis in male partners of unexplained infertile couples undergoing ovarian stimulation and IUI	708	N.A	IIEF	PHQ-9	FertiQol	34.2 ± 24.2 months	N.A	8.9% had IIED score for ED Both fertility-related QOL and depression are strongly and inversely associated with ED
Ramezanzadeh et al. 2006 [62]	Cross-sectional study	200	N.A	Not validated questionnaires	N.A	N.A	3.4 ± 2 yrs	Ovulation induction, IUI, IVF/ICSI	Reduction of sexual desire in 41.5% and satisfaction in 52.5% of cases as compared with recalls before diagnosis of infertility Duration of infertility and duration of desire for a child showed a significant inverse impact on sexual satisfaction (*P* < 0.05)
Muller et al. 1999 [61]	Cross-sectional study about sexual satisfaction	68	N.A	Not validated questionnaires	N.A	N.A	3.1 ± 2.1	N.A (1.6 ± 1.9 yrs tr.duration)	Neither the age of partners, attitudes toward sexuality, treatment duration, duration of the partnership and the duration of the desire for a child, nor andrological findings, had an influence on actual sexual satisfaction
Ozkan et al. 2015 [53]	Prospective longitudinal study	56	48	IIEF-15	BDI score	N.A	> 1 year	IVF	Mild-to-moderate ED was detected in 85.9% of patients in the infertile group
Yikilmaz et al. 2019 [49]	Prospective longitudinal study	193 infertile men 48 male partners having a child after ART treatment	N.A	IIEF-15 PEDT	VAS stress	N.A	27 (12–180 months	Fertility drugs, IUI	ED was found in 35.2% and PE in 21.7% of subjects IIEF-15 scores increased from 16 to 21 (*p* = 0.014) in couples having a baby with ART
Shindel et al. 2008 [64]	Cross-sectional	73	N.A	IIEF-15 SEAR Not validated questionnaires for PE	CES-D	SF-36	N.A	None (before starting ART)	50% of men reported PE. When men reported PE, their partners agreed with the diagnosis in 47% of cases. Female partners of men who did not report PE, reported PE in 11% of cases
Smith et al. 2015 [103]	Cross-sectional analysis	357	N.A	Not validated questionnaires	Not validated questionnaires	Not validated questionnaires	2.1 ± 1.4 yrs in male factor only infertility	No prior IVF/ICSI (previous medical treatment or IUI)	Male partners in couples who perceived isolated male factor infertility have a lower sexual and personal QoL
Kruljac et al. 2019 [65]	Case control study	165 sub-fertile men	199 men	SCS-M	N.A	N.A	Retrospectively cohort had finished infertility treatment, whereas the prospective part had just started the workup	N.A	In HH, statistical significance was seen both in relation to low sexual interest/desire for sex (OR 2.3, 95% CI 1.0–5.5) and for being worried about the size or shape of the penis (OR 3.6, 95% CI 1.3–9.5) Men from infertile couples have an increased risk of symptoms of SD (linked to androgen deficiency)
Elia et al. 2010 [60]	Cross-sectional	156	N.A	Modified IIEF	N.A	N.A	N.A	None (only having sex during the ovulatory period)	The prevalence of SD was significantly higher in the sex-intended-for-reproduction group (23.7%) compared with the control group (before trying to conceive, 5.1%) (*p* < 0.01) or spontaneous sex for pleasure (8.9%) (*p* < 0.01) The total domain scores for sex for reproduction and the specific scores regarding orgasmic function, sexual desire or intercourse satisfaction were significantly lower as compared to spontaneous sexuality or the control group. No differences in erectile function
Gao et al. 2013 [51]	Observational, cross-sectional survey	1468	942	IIEF-5 PEDT IELT	SAS SDS	N.A	N.A	N.A	The incidences of PE and ED in the infertile group were significantly higher than those in the fertile group (PE: 19.01% vs.10.93%, *P* < 0.001; ED: 18.05% vs. 8.28%, *P* < 0.001) IELT and IIEF-5 were negatively associated with anxiety and depression
O’Brien JH et al. 2005 [50]	Case control study	302	60	SHIM ADAM	N.A	N.A	N.A	N.A	38% of infertile men reported significant andropause symptoms and 28% had abnormal SHIM scores The prevalence of ED in infertile men was significantly higher than in the fertile controls (28%, *p* = 0.007)
Saleh et al. 2003 [98]	Cohort observational study about psychosexual problems in men undergoing infertility evaluation	412	N.A	IIEF-5	N.A	N.A	N.A	None	98% (405/412) of patients had normal sexual functions (total IIEF-5 score > 20). Of these, 46 (11%) failed to collect semen by masturbation for the second analysis in 2 weeks upon finding an abnormality of semen parameters (psychogenic SD)
Satkunasivam et al. 2014 [52]	Retrospective study	1750	N.A	SHIM ADAM	N.A	N.A	N.A	N.A	The prevalence of ED and a positive response to the ADAM questionnaire were 30.5% and 45.2%, respectively, unrelated to hormonal changes
Lotti et al. 2012 [55]	Cross-sectional study to assess the prevalence of ED and PE in men seeking medical care for couple infertility	244	N.A	IIEF-15 PEDT NIH-CPSI	MHQ	N.A	N.A	None	ED was found in 17.8% and PE in 15.6% subjects. After adjusting for age, IIEF-15-EFD score was negatively associated with depressive symptoms (MHQ-D score), somatization (MHQ-S score), NIH-CPSI total and QoL subdomain score PEDT score was positively associated with prostatitis symptoms and signs
Song et al. 2016 [58]	Cross-sectional study assessing sexual function and stress in male partners during fertile periods	236	N.A	IIEF-5	VAS stress	N.A	18 ± 7.1 months	None	Stress levels for sexual function were higher during the fertile compared with the non-fertile periods in 46.2% (109/236) of patients. 8.9% reported more than mild-to-moderate ED (IIEF-5 score ≤ 16) and 42% reported mild ED (IIEF-5 score 17–21)
Lotti et al. 2016 [56]	Cross-sectional analysis	448	74	IIEF-15 PEDT NIH-CPSI	MHQ	Chronic Disease Score	N.A	None	Higher prevalence of ED (IIEF-15-erectile function domain score < 26) (18.3% vs 0%; *P* = 0.006) and PE (PEDT score > 8) (12.9% vs 4.1%; *P* = 0.036) in males of infertile couples compared with fertile men. Azoospermic men showed the worst erectile function and general Health
Ma et al. 2021 [54]	Cross-sectional study aiming to evaluate sexual function according to the type of infertility	258 primary infertility and 129 secondary infertility	N.A	IIEF-5	PHQ-9 GAD-7	N.A	N.A	N.A	Higher prevalence of ED in secondary vs. primary infertility (46.5% vs. 26.7%, *P* < 0.001) Primary infertility was an independent risk factor of anxiety

*ED* erectile dysfunction, *IIEF* International Index of Erectile Function, *QoL* quality of life, *PEDT* premature ejaculation diagnostic tool, *VAS* visual analogue scale, *SEAR* Self-Esteem and Relationship Quality scale, *SCS-M* Sexual Complaints Screener for Men, *IELT* intravaginal ejaculatory latency time, *SAS* self-rating anxiety scale, *SDS* self-rating depression scale (SDS), *SHIM* Sexual Health Inventory for Men, *ADAM* Androgen Deficiency in the Aging Male, *MHQ* Middlesex Hospital Questionnaire, *NIH-CPSI* National Institutes of Health–chronic Prostatitis Symptom Index, *PHQ-9* Patient Health Questionnaire, *GAD-7* 7-item Generalized Anxiety Disorder Scale, *IUI* intrauterine insemination, *IVF* in vitro fertilization, *ICSI* intracytoplasmic sperm injection

SD males with infertility often present with erectile dysfunction (ED), defined as the consistent inability to obtain or maintain a penile erection of sufficient quality to permit satisfactory sexual intercourse [47]. The first evidence dates back to 1980, when Berger reported the experience of impotence on 11 patients out of 16 infertile couples [48].

Over time, studies using the popular and well-validated IIEF (International Index of Erectile Function) score have identified a wide variability in the prevalence of ED, ranging from 18 to 85% [49–54].

In two Italian studies by *Lotti *et al. in 2012 and 2016, an ED prevalence of 17.8% and 18.3%, respectively, was reported [55, 56], significantly higher than in men of fertile couples [56]. In the most recent sub-analysis of the *Assessment of Multiple Intrauterine Gestations from Ovarian Stimulation* (AMIGOS) trial, 8.9% of men showed mild-to-moderate ED (assessed prior initiation of fertility treatment), but SD was associated with a worse quality of life and a greater risk of depression [57]. This prevalence, lower than reported in other studies, is possibly due to the study being a secondary analysis of data from a trial with different outcomes and to the almost 21.3% of male partners (192/900 men) not included in the analysis.

The need to plan sexual intercourses with consequential reduction of the spontaneity of sexual pleasure contributes to the onset of SD in the male population. A 2015 Korean study of 236 infertile couples found that 8.9% had more than mild-to-moderate ED (IIEF-5 score ≤ 16), whereas 42% had mild ED (IIEF-5 score 17–21) during fertile periods of the partner, with lower IIEF total score and higher sexual relationship stress during fertile versus infertile time period, confirming that the couple's relationship, in looking for pregnancy, is a key determinant of sexual well-being [58].

In a very large Japanese series of 4220 infertile patients, up to 52.7% of male partners had ED but above all 26.2% had timely ovulatory intercourse failure [59]. The latter data can support the bidirectional relationship between infertility and SD, with ED representing a cause of infertility in planning sexual intercourse for reproduction.

In support to the stressful role of diagnosis and treatment plan of infertility on sexuality*, Elia *et al. have investigated, using the abridged psychometric tool IIEF-5, the sexual health of patients of both sexes for pleasure and intercourse for reproduction (i.e. ovulatory period) [60], showing disorders in sexual intercourse for reproductive purposes in 23.7% of the subjects as compared to 8.9% in sex for pleasure, especially concerning orgasmic function, sexual desire and satisfaction scores (no differences on domain of ED).

Indeed, few studies have investigated the association between infertility and sexual satisfaction in the male population (Table 2) [53, 61, 62]. The reduced satisfaction was closely and highly correlated to the duration of infertility and to the desire for parenthood [62]. Actually, the prevalence of HSDD has been less investigated in male than in female partners, where the desire domain is often very compromised by the infertility-related stress [53, 63].

Finally, only few studies have shown that infertility also involves ejaculation disorders. All studies on this topic agree on an increase in the prevalence of premature ejaculation (PE) in infertile men (12–50%) [51, 64, 65]. In a study carried out in the Italian population, a 12.9% prevalence for PE was found by the *Premature Ejaculation Diagnostic Tool* (PEDT) among infertile males, but prevalence was even higher in the subgroup of azoospermic men [56]. Additionally, ED and PE could be associated, with each one contributing to the development, or progression from subclinical to overt, of the other [66], therefore, providing additional confounding factors to the exact pathogenetic mechanisms associated with infertility. For all these reasons, the most recent guideline on PE management published by the Italian Society of Andrology and Sexual Medicine (SIAMS) suggested investigating sexual function as well as psychological health status of male patients of infertile couples [67].

### Sexual dysfunctions: a view of couple

Infertility makes a real challenge to the sexual life and SD are an emerging paradigm as a typical “couple disorder” [68], especially in the setting of infertility, where it constitutes a broader dimension that can be summarized in the concept of ISS.

Clearly, in the infertile couple, the intercourse is often scheduled and deprived of spontaneity. In the specific setting of the therapy of infertility, the purpose of the sexual intercourse is the conception of a child with the consequential de-eroticization of the sexual act. An alteration in the couple communication can occur in the presence of conflicts, along with adjustments of the relationships with a role of strength in favor to the fertile partner. In this sense, psychological distress, marital relationships and sexual satisfaction are closely linked in infertile couples [69].

For this reason, the evaluation of sexuality should consider the couple as a whole. The majority of studies included the perspective of a single partner, without considering that both partners’ responses to infertility interact and influence each other’s psychological and sexual adjustments.

Several studies have contextualized the assessment of sexual function in the couple (Table 3). In 200 infertile couples whose infertility factor was 62% female and 17.5% idiopathic, a significant reduction in the FSFI score was found, particularly in the domains of satisfaction, orgasm and pain. Actually, their husbands had a significantly higher prevalence of ED (52.5%) and PE (23.5%) tested by SHIM score (Sexual Health Inventory for Men, a validated, 5-items version of IIEF score), a condition that was not the cause of infertility but contributed significantly to the worst sexual health of women [70]. Similarly, a positive correlation between FSFI and IIEF scores was found among partners of infertile couples [27]. At the same time, SD in the female partner were a positive predictor of ED of the male within couple [71]. These findings support the emerging paradigm of ISS as a sexual and relationship disorder of infertile couples and emphasize the importance of screening both partners before planning an ART treatment for infertility.

**Table 3:** Studies of female and male Ssxual dysfunctions within infertile couples (analysis and comparison of both female and male partners)

Study	Infertile women/men (couples)	Fertile women/men (couples)	MSD assessment	FSD assessment	Psychological assessment	QoL assessment	Infertility duration	ART treatment	Results
Benazon et al. 1992 [82]	Longitudinal study of 12 months duration on 165 couples attending fertility clinic 117 couples never became pregnant during infertility treatment process while 47 couples became pregnant during the investigation		ISS	ISS	PSS	N.A	N.A	N.A	Significant increase in stress and decrease in marital functioning were experienced in couples as the treatment investigation progressed Greater levels of marital distress were observed in couples that did not conceive Non-pregnant women experienced higher levels of stress and lower levels of sexual satisfaction than women who became pregnant
Monga et al. 2004 [4]	18	12 seeking surgical sterilization	IIEF-15	BISF-W	MAT	QWB-SA	N.A	N.A	83% of couples reported feeling societal pressures to conceive Infertile women reported poor marital adjustment and QoL compared with controls. Men experienced less intercourse satisfaction
Daniluk et al. 1988 [81]	43 infertile couples	N.A	ISS	ISS	SCL90 MAT	RCS	N.A	None	Significant distress was experienced by couples at the time of diagnosis. Relationship quality did not appear to deteriorate as a result of the medical investigation
Shindel et al. 2008 [71]	121 infertile couples	N.A	IIEF-15	FSFI	CES-D	SF-36	10 ± 1.3 months	N.A	FSFI score were significant predictors of IIEF-Erectile Function Domain scores (*p* < 0.01) 11% and 12% of male partners reported moderate or severe depression
Khademi et al. 2008 [90]	100 infertile couples	N.A	IIEF-5	SFQ	N.A	N.A	5.3 ± 3.7 yrs	N.A	93% of women were at risk of FSD, with the arousal domain as the most affected 61.4% of men had mild-to-moderate ED (only 2% severe ED)
Shoji et al. 2014 [93]	93 infertile coupes	92 couples with recent pregnancy	GRISS	GRISS	BDI	N.A	40 ± 31.9 months	19.4% none 68.8% timed intercourse 36.6% IUI 28% IVF/ICSI	Sexual satisfaction deteriorated with therapeutic interventions Therapeutic interventions such as timed sexual intercourse and ART were emotionally stressful for infertile couples, with lower sexual satisfaction in this group than in couples achieving spontaneous pregnancy
Yangin et al. 2015 [77]	102 infertile couples	N.A	GRISS	GRISS	BDI	N.A	10% < 2 yrs 57.8% 2–5 yrs 24% 6–9 yrs 16.7% > 10 yrs	33.3% none 66.6% ≥ 1 ART treatment (not specified)	Higher prevalence of SDs in women than in men. Infertile couples experienced a higher number of SDs during the infertility treatment process
Lee et al. 2001 [88]	138 infertile couples (classified in female, male, mixed and unexplained factor infertility)	N.A	CIFQ SSQ	CIFQ SSQ	MSQ	N.A	> 1 year	Ovulation induction, IUI, IVF/ICSI with embryo-transfer	Female members of couples in which both partners were infertile expressed less marital and sexual satisfaction than their husbands. No differences in marital and sexual satisfaction were found between wives and husbands with unexplained infertility In infertile couples, female factor of infertility is significantly associated to infertility distress and both marital and sexual dissatisfaction in women
Drosdzol et al. 2009 [89]	206	190	ISS	ISS	IMS (Polish Version)	N.A	36.5 ± 40.1 months	N.A	Diagnosed male factor and infertility duration of 3–6 years were connected with the highest relationship instability and the lowest sexual satisfaction both in female and male
Marci et al. 2012 [83]	60 (Group A = 30 recently diagnosed infertile couples and Group B = 30 already undergone IUI)	52	IIEF	FSFI	ACL	N.A	Group A: diagnosed within 2 months Group B: N.A	IUI	Men of all the three groups scored higher in both questionnaires (sexual satisfaction, desire and orgasm) than their female partners Infertile patients experienced great stress even in the very first phase of treatment: just diagnosed male partners obtained lower scores in all the subscales. Infertile women of both groups showed an impairment of sexual arousal, satisfaction, lubrification and orgasm when compared to fertile ones
Purcell-Levesque 2018 [75]	45 couples	N.A	Arizona Sexual Experiences Scale Global Measure of Sexual Satisfaction	Arizona Sexual Experiences Scale Global Measure of Sexual Satisfaction	Experiences in Close Relationships scale	N.A	4.49 ± 3.36 yrs	N.A	Attachment-related anxiety positively predicted ED in men (95% CI [0.01, 0.59]), and lubrication problems in women (95% CI [0.14, 0.75]) Men’s avoidance positively predicted their partner’s difficulty to reach orgasm
Yeoh et al. 2014 [69]	150 females and 119 males	N.A	IIEF	FSFI	N.A	N.A	3.8 ± 2.8 years	IVF or ICSI (25.3%)	11.3% of women classified as having sexual dysfunction A strong correlation between male and female sexual function was observed
Zare et al. 2017 [37]	110 couples	110 couples	GRISS–M	GRISS–F	N.A	N.A	4.85 ± 3.53	41.8% of couples had previously undergone ART	Infertile men reported more problems in relations, impotency and PE compared to fertile men. Men in both fertile and infertile group reported more sexual problems than women
Gabr et al. 2017 [70]	200	200	SHIM	FSFI	N.A	N.A	N.A	23% of infertile group had previous IVF/ICSI	FSD was found higher in infertile vs control group (47% vs 30%). Total FSFI, orgasm, satisfaction and pain sexual domain scores were significantly lower in the infertile group whereas the desire, arousal and lubrication scores were not significantly different between groups In husbands, the prevalence of ED (52% vs 19%) and PE (23.5% vs 10%) was significantly higher in the infertile men
Cocchiaro et al. 2020 [79]	162	N.A	SEIq	SEIq	SEIq	N.A	3.1 ± 2.5	N.A	The incidence of SDs was 10% in men and more frequent in women (29%) (*p* < 0.01). Infertility diagnosis changed the sexual desire and habits in 50–60% of the couples, in women more than in men

*FSD* female sexual dysfunctions, *MSD* male sexual dysfunctions, *ED* erectile dysfunction, *PE* premature ejaculation, *QoL* quality of life, *ISS* Index of Sexual Satisfaction, *PSS* psychological state of stress, *IIEF* International Index of Erectile Function, *QWB-SA* Quality of Well Being Self-Administered, *SCL* Symptom Check List, *RCS* Relational Coordination Scale, *BDI* Beck Depression Inventory, *CIFQ* Chinese Infertility Questionnaire, *SSQ* Sexual Satisfaction Questionnaire, *MSQ* Marital satisfaction Questionnaire, *IMS* Index of Marital Satisfaction, *ACL* Adjective Check List, *SHIM* Sexual Health Inventory for Men, *CES-D* Center for Epidemiological Studies Depression Scale, *FSFI* Female Sexual Function Index, *BISF* Brief Index of Sexual Functioning, *SFQ* Sexual Function Questionnaire, *GRISS* Glombok-Rust Sexual Status questionnaire, *SF-36* Short Form 36, *MAS* Marital Adjustment Scale, *BDI* Back Depression Inventory, *FPI* Fertility Problem Inventory, *SEIq* Sexuality and Emotions in Infertility questionnaire, *IUI* intrauterine insemination, *IVF* in vitro fertilization, *ICSI* intracytoplasmic sperm injection

Personalities, as measured by the attachment styles [72, 73], and psychopathological traits, such as anxiety and depression [74], are dramatically able to influence sexual behaviors in both sexes. A recent study investigated the sexual function in couples undergoing infertility treatments in association with attachment insecurities (anxiety, avoidance) [75]. This is a further aspect to consider in the complex couple’s relationship within the infertility setting: anxiety in woman was associated with difficulties in lubrication, while difficulty in reaching female orgasm was related to avoidance by the male partner. Undoubtedly, medically prescribed sexual intercourse along with an unresponsive or insensitive male partner, may relate to a female sexual pleasure that is felt less of a priority.

A focus point in assessing sexuality within the couple, is the gender difference in the experiential and personal response to the childlessness. The more severe psychological impact of infertility on the female partner has often been speculated [76–79], although a lot of men experience increasingly prevalent SDs in response to the diagnosis and treatment of infertility [80].

Early studies, over 30 years ago, pointed out that women experienced higher levels of stress than male subjects, already at the time of diagnosis of infertility, with an interaction between gender and sexual satisfaction [4, 81, 82]. This finding can be partially framed by the consequences of treatments for infertility that are usually more intrusive for women and mainly impact on female quality of life [82, 83]. Moreover, mood disorders are more prevalent in women: clinical depression occurs in 26.2% of women and 9.2% of men undergoing ART procedures, while anxiety may be encountered in 14.8% of women and 4.9% of men [78, 84]. Motherhood is believed to be a perceived essence of a woman’s identity and an identity as infertile woman receives a huge emotional investment [4, 85]. The loss of self-esteem and sexual relationship satisfaction occurring with the failed search for fertility, seems to be more pronounced in the female partner [86]. Particularly, sexual satisfaction is adversely affected by the consequences of infertility, such as poor self-confidence and depression, which lead to couples’ burnout, intended as an emotional and physical exhaustion caused by the incompatibility between expectations and reality. Sexual burnout has a significant impact on both general and somatic exhaustion and distress, and it is experienced more by infertile women than their male partners, being associated to psychological disorders, marital issues and dissatisfaction [87].

However, in couples undergoing infertility treatment the male partner may experience a loss of potency, mostly exacerbated by and during scheduled sexual intercourse [80]. It has been demonstrated that men who experience stress and SD from infertility and its diagnostic-therapeutic procedures have significantly low sperm parameters. The reduction of male stress associated with ART procedures represents an essential component of infertility management, also to reduce the vicious circle related to sperm collection and improve sperm parameters [80].

The etiology of the infertility is another important factor in assessing gender differences in distress and sexual satisfaction: in couples where both partners were infertile, only women experienced less marital and sexual satisfaction, while the female infertility factor leads to higher self-esteem distress only in woman as compared to husbands, with no differences either in sex or in distress in male partners regardless the infertility diagnosis [88]. However, there are no univocal results [89, 90].

Interestingly, psychological and sexual attitude may change through the course of infertility treatment. It has been shown that these components did not deteriorate through the diagnostic plan (from diagnosis and in the following 6 weeks), even if the highest level of stress was found at the time of the initial assessment [81]. In contrast, others speculated an effect of the treatment steps on sexual function. Considering the assessments of both members of the couple before and after intrauterine insemination (IUI), in men only the ED subscale had a lower score at the time of diagnosis (therefore, before treatment), which could be linked to the performance of the diagnostic exams. Again, the gender difference was reflected in a lower score of almost all FSFI domains in both treatment phases (before and after IUI) as compared to fertile women [83].

In point of fact, the recruitment of both partners should be encouraged in future studies to assess how both members contribute to the couple’s sexual adjustment in the context of fertility treatment settings. The use of validated tools, that offer a framework to contextualize the sexual and psychological function into the couple, should be encouraged.

## Sexual function in couples seeking fertility treatment by ART

Sexual dysfunction in couple undergoing ART is a relatively new topic, only recently explored by scientific literature. In fact, most studies on the impact of infertility in sexuality have involved couples seeking fertility care at the first assessment in infertility clinics, with no systemic evaluation of the impact of the infertility treatment program on sexual health [90, 91]. Although a substantial agreement was detected in the recent literature, we found significant heterogeneity among the experimental protocols, mainly because of the different aims and methodologies adopted to assess sexual function (Tables 1, 2, 3).

Couples requesting ART may be sexually dysfunctional either for the mentioned psychological pressure of childlessness (and for all the reasons seen before, whereby SDs represent a couple disorder within the infertility setting) or for a reaction to failed infertility treatments. Interestingly, couples undergoing ART procedures, such as IVF, reported that sex became less enjoyable after infertility treatments [92]. In a Japanese study, in 92 couples who started therapeutic interventions such as IUI and IVF, the needed, obvious manipulation of the sexual setting significantly produced infrequency, non-sensuality, non-communication, male dissatisfaction and male avoidance [93].

Infertile couples undergoing ovulation induction treatment and timed intercourse, followed by IUI on the third month, have been investigated by *Bayar *et al. [94]: in this study, both partners experienced an increase in the prevalence of SD—from 60 to 72% in women and from 34 to 48% in men. In the male partners there was an increase in HSDD, while in women all domains were affected, but especially disorders of orgasmic satisfaction and arousal [94]. It is likely that physically disturbing procedures, the sense of being “monitored”, the need of “having sex by the clock”, the loss of the eroticization of intercourse (first aimed to conception and afterwards deprived of its main purpose) affect sexual self-image and desire. A negative experience of repeated treatment failures may also contribute to the increase of SD. In this study, however, the FSFI score did not differ before or after IUI treatment in women, although it remained significantly lower than in fertile couples, while the IIEF score in men was significantly lower at the time of diagnosis. The authors speculated that the sample of patients were candidates for IUI, therefore, they suffered from a moderate infertile condition that did not require complex techniques, and could still have a more optimistic view of their situation and less psychological and sexual distress [83].

The ART method has an effect on the frequency of sex. IUI couples calculate more often the space between relationships to enrich the quality of the sperm than couples under IVF)/intracytoplasmic sperm injection (ICSI). In addition, couples under IUI have more sexual intercourses during treatment periods than couples who are on IVF or ICSI [95].

However, this result highlighted the different factors of infertility treatment that affect the prevalence and type of SD either in men or in women.

### Male sexuality under ART

At the beginning of infertility treatment, men often experience a feeling of stigmatization because of a perceived lack of masculinity, which is possibly even more challenging than the therapy for infertility itself [96]. Men tend to adjust their sexuality to the project of conception and often mistake the ability to conceive with virility [95]. Moreover, men often experience more difficulties then their female partners to accept an ART program, just because they perceive it an intrusion into their virility [97]. The requirements of medically assisted procreation—i.e. abstinence period, collection of sperm from masturbation in a cramped room—also contribute to the difficult acceptance of the condition.

An Italian survey found high prevalence of male SD among men undergoing ART, with 56.2% reporting ED, 25% PE and 18.7% HSDD [6]. In a Japanese study in 92 couples who started ART treatment, such as IUI and IVF-ET, the sexual satisfaction of male partners was significantly lower than female partners in those aged 30 years or older [93].

During ART, medical investigations and female expectations may lead to SD in the male partner [97]. Men often feel at unease with their involvement as “sperm donors”: 11% of men cannot provide the sample on command, following transient ED or orgasmic disorder, in particular after detection of an abnormality in the results of their previous semen analysis [98]. Producing semen in the IVF clinic may be stressful and perceived as intrusive, therefore, contributing to the onset of ED or worsening of subclinical symptoms [66, 99], to a greater extent in subjects with a longer duration of infertility and with increased levels of anxiety [96, 98]. In infertile men, the close association between ED and psychological burden has been demonstrated (especially anxiety and depression) [100].

In infertile men seeking fertility treatments, sexuality is closely linked to the outcome of procreation. A study of 141 Turkish infertile couples undergoing IVF treatments showed that in couples who had obtained a child through ART, the IIEF-15 score increased significantly from 16 to 21, demonstrating that experiential anxiety could contribute to the onset ED. Instead, no effect was shown in the prevalence of PE tested with PEDT tool. The successfulness of ART treatment and the pregnancy achievement could have a reducing effect on sexual relationship stress [49].

A possible significant contribution on male SDs and on marital relationships is the duration of treatment or the number of ART cycles, but unfortunately this parameter has been seldom investigated. A study reported that in newly diagnosed and treated infertile patients (within 3 months), sexual satisfaction was reduced compared to those diagnosed with longer infertility; however, the duration of treatment did not correlate with a worsening of sexual satisfaction [62]. Another study in Turkish infertile population undergoing ART found no relationship between SD and sexual satisfaction on one hand, and duration of infertility, duration of treatment and number of ART treatments on the other hand [77].

More extensive research on all these topics is warranted.

### Female sexuality and ART treatments

Most of the studies on sexuality in couples undergoing ART have focused on the female component. During ART, especially in women, sexual interest, frequency of intercourse and sexual satisfaction decrease. Every month there is a distressing wait for the result of treatment. In women every failed cycle may stress the symbolic or real loss of a child, worsen self-esteem and generate feelings of anger, depression, emptiness, sadness and guilt.

The Italian Society of Andrology and Sexual Medicine (SIAMS) found that 43.7% of women seeking fertility treatment had HSDD and 12.5% experienced dyspareunia [6]. Such a high prevalence of SD in women undergoing ART is confirmed by studies carried out in other populations: in Iranian women referred to an infertility clinic, albeit with a non-validated tool, 55% had impaired sexual function and sexual satisfaction. Up to 41.6% reported a change in the frequency of sexual intercourse and more than 50% reported experiencing anxiety during an intercourse after starting infertility treatments [101]. This result confirms the impact of both the diagnosis of infertility and therapeutic procedures and their failures on psycho-sexuality of the female [40].

The evaluation of homogeneous populations, such as those undergoing specific ART treatments, can help to identify the specificity of the FSD, and consequently to set in the sexual, psychological and quality of life comprehensive aspects. Since IUI is usually performed at early stages of infertility treatment, their cycles may appear be less stressful when compared to IVF or ICSI. However, patients undergoing IUI experienced lower sexual function (significantly reduced FSFI) especially in the domains of arousal, desire and satisfaction, as well as a negative effect on the quality of life [102].

Women undergoing IVF may be at higher risk for SD. In 136 women undergoing IVF within the past year or currently on IVF program, a significantly less desire for sex, more difficulties achieving orgasms, less frequent sexual activity, less satisfaction with sex, and poorer overall sexual function were reported as compared with the non-clinical cohort. The most prevalent sexual problem was lack of sexual interest or desire, with over 30% of cohort reporting “no desire”, while inability to orgasm was the second most commonly reported problem. These sexual concerns resulted to be predictive of poorer fertility-related quality of life, assessed by *Fertility quality of life tool* (FertiQoL), an internationally validated instrument to measure quality of life in individuals experiencing fertility problems. The first interesting aspect was the lack of statistical relationship between sexual function, the duration of infertility and the number of the IVF cycles. On the other side, 23% of women reported not being sexually active in the previous month, because of low desire, anxiety about sexual performance, vaginal dryness, but also for ART treatment-related requirements, such as the coached abstinence, or psychological burden [103]. For all these reasons, women undergoing IVF have a higher risk of SD.

More recently, other two works investigated the effects of the IVF program on female sexuality. A Turkish prospective study found a very high prevalence of SD (above 90%), measured by FSFI, among women undergoing IVF treatment, independently of the causes of infertility (idiopathic or by poor ovarian reserve) [104]. The author speculated that the high rates in the study were partially due to religious and cultural issues; moreover, all the patients were scheduled for IVF, with a prolonged history of medication resulting in a longer period of discomfort and a huge anxiety related to the highly intensive and challenging nature of the treatment. Another study, again on a Turkish population, confirmed that sexual function was adversely affected by the long duration of infertility and the increase in the duration of treatment [41]. They stressed the need to consider sexual health in couples undergoing IVF, because the stress resulting from the treatment overlapped the anxiety related to infertility.

The rate of FSD in couples undergoing IVF was lower in an Italian study, where 30% of 269 patients reported an FSFI score ≤ 26.55, as compared with other studies [105]. In this survey, women had been enrolled on the day of the oocyte collection, therefore, in coincidence with the peak physical and psychological effects of the treatment itself. Female sexual function in this special population was associated with specific psychological risk factors: all FSFI domains (desire, arousal, lubrification, pain, orgasm and satisfaction) were affected and inversely related to FPI score, particularly infertility-related social, relational and sexual concerns. Therefore, the cause of infertility (male or female) and the number of previous IVF cycles seems here not really able to affect the female sexual function. It may be thought that infertility itself is not directly related to SD, but also that female sexual function is mainly associated with the psychological concerns linked to infertility. Social concerns may be generated by the above-mentioned social pressure about conception or by the time spent with couples who have a child. To prevent the psychological burden of couples and the (rarely mentioned and rarely studied) potentially high risk of drop out from treatment, it is necessary that all these individual, relational, social and specifically sexual wellness aspects are not neglected. Addressing sexual health in several (if not all) medical contexts has an unique and peculiar role in motivating patients to follow challenging diagnoses and therapies [19].

Very few studies evaluated the presence of genito-pelvic pain/penetration disorder (GPPPD) in women under ART, even if they are the expression of the mutual connection between infertility and FSD. Vaginismus prevents both the intercourse and the ability to conceive and expectations of women’s sexuality and fertility may particularly impact on the sufferers [106, 107]. Out of the 236 infertile women evaluated by *Bakhtiari *et al*.*, 28% suffered from dyspareunia and 15.5% from vaginismus [101]. Only one prospective observational study investigated the prevalence of vaginismus in the female population undergoing an IVF/ICSI procedure, identifying few cases but focusing on some clinical conditions that should be detected and managed during infertility treatment [108]. Vaginismus seems to be less prevalent than other sexual dysfunctions but psychological variables are the most responsible factors for vaginismus [109–111]. It is likely that often women might not be aware of their disorder, which might lead to a low prevalence rate [112].

Finally, limited studies were conducted in couples undergoing heterologous fertilization and oocyte donation. Heterologous artificial insemination introduces a third figure (the donor) into the dynamics of the couple, which may impact on the sexual life. Although particularly important, this aspect has been poorly studied yet. Women awaiting oocyte donation may experience emotional and sexual impairments. In an American study, one-third of women who underwent IVF with oocyte donation were depressed and 46% had an FSFI score indicative of SD risk [113].

Evidence proves that the type of protocol may influence the sexual desire, and not exclusively in women: couples enrolled in IUI–IVF have more sexual desire disorders than couples enrolled in ART procedure with donor. However, couples with an intra-marital reproduction project (IUI and IVF) more often recognize that they seek pregnancy more than sexual pleasure with respect to couples under heterologous IVF [95]. Overall, couples in homologous ART were shown to have higher depressive state, anxious symptoms, general infertility-stress and specific infertility-related sexual concerns than the heterologous group [78], with a significant impact also on the quality of life [114]. All these results may be explained by the higher expectation of the couples undergoing homologous ART that might feel themselves more responsible for the pregnancy outcome. Furthermore, in heterologous ART procedures, male gender has been associated with lower anxious symptoms, and in both partners infertility-stress dimensions were less related to depression and anxiety [78].

## Sexuality assessment in ART protocol: an unmet need

Infertile couples seeking infertility care should be walked through sexual functioning. In particular, their emotional, mental and sexual problems must be addressed within infertility treatment programs [79].

There are many factors that make the assessment of sexual function and the management of ISS a necessity in couples undergoing ART.

First, procreation disconnected from sexuality and (natural) fertility redraws the bounds of the couple, through the presence of third figures, acting on both diagnosis and therapy of infertility, extending to them the perimeters of intimacy.

Couple closeness and intimate behaviors could be impaired by diagnostic procedures, intrusiveness of treatments and by the complex medical prescriptions. Moreover, the not unusual ART failures, which exceeds two-thirds of the aspiration cycles [115], can further destabilise the couple's solidity [116].

The infertility-related distress, especially for women, affects the quality of sexual function, while sexual concerns may impact couple sexuality during ART treatment [105]. This emotional stress can be so strong that in a high percentage of cases it leads to drop out since the first cycle of treatment [117]. This may explain why up to 24% of couples, with unexplained or mild male infertility, are able to naturally conceive after ART failures [118]. Therefore, the close relationship between emotional, psychological and sexual issues can impair the treatment outcomes. In anxious and depressed women, fertility treatments seem to be less effective. Among infertile women undergoing their first cycle of IVF, those with a recent diagnosis of anxiety or depression were 40% less likely to conceive (OR 0.58) as compared with women without psychological impairments [119]. In a national registry of more than 40.000 ART-treated women in Denmark, a diagnosis of depression was associated with a significantly lower number of ART cycles and a lower mean number of ART live births [120].

The psychosocial implications of infertility and ART are substantial: particularly, the couple’s sense of self-identity and personal acting, mental wellness, sexual and marital relationships, reproductive efficiency, compliance with treatment and pregnancy outcomes are all impacted by psychological influences, which in turn are integrated into the couple relationship [121]. The unique psychosocial set of ART treatment for infertility may directly interfere with a couple’s usual pattern of sexual behavior, resulting in SD for both partners [122]. While there is at the present date no study investigating the topic using validated tools, such as the Orgasmometer [123, 124], it is likely that orgasmic function of infertile couples is also impaired, due to a de-eroticization of the sexual act.

An integrative look at the medical and non-medical (psychological and social) factors that contribute to sexual problems is mandatory both at the first stage of infertility diagnosis, and during the most advanced stages of treatment and ART procedures.

Psycho-sexological counselling is essential for the infertility treatment to identify couples at risk of developing psychic and/or sexual dysfunctions, aiming to reduce anxiety, to explore emotional aspects and address the psychological, relational and sexual problems related to ART process and its possible failure, even to accompany parenthood in case of a positive outcomes. The negative impact of ART treatments on sexual life with the possible reduction of sexual encounters and the effect on sexual desire may trigger transitory SD, possibly transitioning to permanent SD [15].

The medical and therapeutic program should encompass an integrated approach to address the stress associated with treatment of infertility, along with a sexological assessment through standardized psychometric tools (IIEF and PEDT for men, FSFI for women and Orgasmometer for both).

The management of ISS will help to bring again in the couple’s dimension the emotions and intimacy decontextualized by ART procedures. Moreover, the assessment of psychological health and sexual wellness is fundamental to plan a possible pharmacological intervention. For example, in men seeking fertility care, ED is the most frequently diagnosed SD [6, 48], for which is necessary to consider both organic and non-organic comorbidities [125]. If psychological components prevail, low-dose PDE5i along with psychological therapy may be effective [48, 126]. Sildenafil administration before semen collection for IUI or planned intercourse for a postcoital test is shown to be effective in reversing stress-induced transitory ED and improving seminal parameters, such as the percentage of spermatozoa with linear progressive motility, as well as the number of spermatozoa penetrating the cervical mucus [80]. In organic ED, PDE5i such as sildenafil or avanafil should be prescribed [127–130]. It is also worth mentioning that in some cases the diagnosis of infertility can felicitate the progression from subclinical to overt ED [99] or to overt PE [66], as depression and anxiety could be the proverbial “straw that breaks the camel’s break” in a subject with marginally impaired control over erection or ejaculation.

Instead, lifelong and acquired PE [131] are most likely to benefit from combination therapy of pharmacological treatment with short-acting serotonin re-uptake inhibitor dapoxetine and psychosexual behavioral therapy [48, 67, 132]. Also PDE5i may be useful for treatment of PE that have showed to prolong IELT with acceptable adverse events [133].

In female partners undergoing ART treatment, the presence of dyspareunia, vulvodynia and vaginismus must be assessed, because psychosexual factors are present in these sexual pain-related disorders. Vulvodynia and dyspareunia can be frequent in young women, especially if subjected to intrusive genital procedures: they can be caused by infections (i.e. candidiasis), vulvar dermatoses, irritations from excessive hygienic habits, or by lubrication disorders frequently associated with arousal dysfunction. Vaginismus is one of the least considered dysfunctions, frequently diagnosed after a complaint about infertility instead of sexual intimacy [107]. It should be always taken into account that an ART program implicates vaginal manipulation during transvaginal ultrasound (TVUS) examination in ovarian stimulation, oocyte retrieval and embryo transfer, entailing a challenge to these patients [108].

Disorders of female sexual interest and arousal must be managed through sexual education, psychological couple support, psychotherapy and, in peculiar sociocultural contexts, even with meditation training or acupuncture to further reduce anxiety and depression [134, 135].

A multidisciplinary team is essential within a fertility care setting, which considers a patient-centered and couple-centered perspective to manage the ISS, along with intra-cycles interventions aiming at both ART success and psychosexual and marital wellness.

## Conclusion

Infertility constitutes a challenge for the sexual life, with SD emerging as a typical paradigm for “couple disorder”, particularly in the setting of infertility in which can be framed as the concept of ISS. The merit of this new taxonomic category could be found in the identifying the peculiarity of sexual dysfunction due to infertility—such as HSDD or ED, and, vice-versa, of infertility due to sexual dysfunction—such as HSDD or vaginismus. Moreover, the ISS stresses the need of considering fertility and sexuality as a strict clinical unity, promoting the dialogue, not yet strong enough, between experts in reproductive and sexual medicine(s). In fact, either different cultural habits in the examined patient cohorts, or the lack of standardized tools for sexuality assessment in sterility care setting, or the different comorbidities that overlap throughout the course of infertility treatment, make the real dimension of psycho-sexological impact of infertility difficult to assess, understand and to treat. Doctors exclusively focused on the lack of fertility itself and to the technical aspects of the diagnosis and treatment may easy lose this crucial part of the issue.

Unavoidably, psychological and sexological attitudes change through the course of the infertility diagnostic program, with different implications for the members of the couple. When the couple needs recourse to ART, additional factors may be involved such as either intrusiveness of the procedures, or perception of the disconnection between sex and reproduction, de-eroticization of the sexual intercourse, or finally, increased psychological pressure in case ascribable to procedural failures. Women, often more involved in ART procedures regardless of the cause of infertility, are likely to be particularly affected in their psycho-sexological life side, especially if second level techniques (IVF/ICSI) are requested.

Extensive evidence makes assessment of the psycho-sexological function mandatory in couples under ART. Rewiring the couple dimension with loss of intimacy and involvement of third figures, and the mutual relationship among emotional stress, sexual function and outcomes of ART procedures, may contribute on higher percentage of treatments’ failures or drop out of the couples. The future challenge is to support both partners, to design a couple-centred approach in the infertility treatment plan, grounded on a multidisciplinary team that guarantees a comprehensive management of ISS, as all the emotional, relational and, inevitably, sexual aspects of couples seeking parenthood.

## References

[1] Fassino S, Pierò A, Boggio S (2002). Anxiety, depression and anger suppression in infertile couples: a controlled study. Hum Reprod.

[2] Peterson BD, Sejbaek CS, Pirritano M, Schmidt L (2014). Are severe depressive symptoms associated with infertility-related distress in individuals and their partners?. Hum Reprod.

[3] Sansone A (2021). Medice, cura te ipsum: a first-person experience with male infertility. Andrology.

[4] Monga M, Alexandrescu B, Katz SE (2004). Impact of infertility on quality of life, marital adjustment, and sexual function. Urology.

[5] Haimovici F, Anderson JL, Bates GW, et al (2018) Stress, anxiety, and depression of both partners in infertile couples are associated with cytokine levels and adverse IVF outcome. Am J Reprod Immunol 79:. https://doi.org/10.1111/aji.12832 10.1111/aji.12832 29528174

[6] Ciocca G, Limoncin E, Mollaioli D (2015). SIAMS survey on sexological screening during the assisted reproductive technologies in Italy. J Endocrinol Invest.

[7] Zegers-Hochschild F, Adamson GD, de Mouzon J (2009). International committee for monitoring assisted reproductive technology (ICMART) and the world health organization (WHO) revised glossary of ART terminology, 2009*. Fertil Steril.

[8] Mascarenhas MN, Flaxman SR, Boerma T (2012). National, regional, and global trends in infertility prevalence since 1990: a systematic analysis of 277 health surveys. PLoS Med.

[9] Lenzi A, Lombardo F, Salacone P (2003). Stress, sexual dysfunctions, and male infertility. J Endocrinol Invest.

[10] Rowland DL, Jannini EA (2020) Cultural Differences and the Practice of Sexual Medicine, Springer I

[11] Pacheco Palha A, Lourenço MF (2011). Psychological and cross-cultural aspects of infertility and human sexuality. Adv Psychosom Med.

[12] Rutstein SO, Shah IH (2004) Infecundity, infertility, and childlessness in developing countries. DHS Comparative Reports 9. DHS Comp Reports 9:pages 13–50. https://doi.org/PN-ADB-836

[13] Burri A, Graziottin A (2015). Cross-cultural differences in women’s sexuality and their perception and impact of premature ejaculation. Urology.

[14] Gremigni P, Casu G, Mantoani Zaia V (2018). Sexual satisfaction among involuntarily childless women: a cross-cultural study in Italy and Brazil. Women Heal.

[15] Piva I, Lo Monte G, Graziano A, Marci R (2014). A literature review on the relationship between infertility and sexual dysfunction: does fun end with baby making?. Eur J Contracept Reprod Heal Care.

[16] Carosa E, Sansone A, Jannini EA (2020). Management of endocrine disease: female sexual dysfunction for the endocrinologist. Eur J Endocrinol.

[17] Maseroli E, Fanni E, Cipriani S (2016). Cardiometabolic risk and female sexuality: focus on clitoral vascular resistance. J Sex Med.

[18] Mollaioli D, Ciocca G, Limoncin E (2020). Lifestyles and sexuality in men and women: the gender perspective in sexual medicine. Reprod Biol Endocrinol.

[19] Jannini EA (2017). SM = SM: the interface of systems medicine and sexual medicine for facing non-communicable diseases in a gender-dependent manner. Sex Med Rev.

[20] Unuane D, Tournaye H, Velkeniers B, Poppe K (2011). Endocrine disorders & female infertility. Best Pract Res Clin Endocrinol Metab.

[21] Zhao S, Wang J, Xie Q (2019). Is polycystic ovary syndrome associated with risk of female sexual dysfunction? A systematic review and meta-analysis. Reprod Biomed Online.

[22] Wiegel M, Meston C, Rosen R (2005). The female sexual function index (FSFI): cross-validation and development of clinical cutoff scores. J Sex Marital Ther.

[23] Isidori AM, Pozza C, Esposito K (2010). Development and validation of a 6-item version of the female sexual function index (FSFI) as a diagnostic tool for female sexual dysfunction. J Sex Med.

[24] Rooney KL, Domar AD (2018). The relationship between stress and infertility. Dialogues Clin Neurosci.

[25] Slade P, O’Neill C, Simpson AJ, Lashen H (2007). The relationship between perceived stigma, disclosure patterns, support and distress in new attendees at an infertility clinic. Hum Reprod.

[26] Ozkan B, Orhan E, Aktas N, Coskuner ER (2016). Sexual dysfunction and depression among Turkish women with infertile husbands: the invisible part of the iceberg. Int Urol Nephrol.

[27] Nelson CJ, Shindel AW, Naughton CK (2008). Prevalence and predictors of sexual problems, relationship stress, and depression in female partners of infertile couples. J Sex Med.

[28] Suna KK, Ilay G, Aysenur A (2016). Effects of infertility etiology and depression on female sexual function. J Sex Marital Ther.

[29] Pakpour AH, Yekaninejad MS, Zeidi IM, Burri A (2012). Prevalence and risk factors of the female sexual dysfunction in a sample of infertile Iranian women. Arch Gynecol Obstet.

[30] Lo SS tsing, Kok WM (2016) Sexual functioning and quality of life of Hong Kong Chinese women with infertility problem. Hum Fertil 19:268–274. Doi: 10.1080/14647273.2016.1238516 10.1080/14647273.2016.1238516 27706954

[31] Salomão PB, Navarro PA, Romão APMS (2018). Sexual function of women with infertility. Rev Bras Ginecol e Obstet.

[32] Hentschel H, Alberton DL, Sawdy RJ (2008). Sexual function in women from infertile couples and in women seeking surgical sterilization. J Sex Marital Ther.

[33] Oindi FM, Murage A, Lema VM, Mukaindo AM (2019). Association of female sexual dysfunction and fertility: a cross sectional study. Fertil Res Pract.

[34] Furukawa AP, Patton PE, Amato P, et al (2012) Dyspareunia and sexual dysfunction in women seeking fertility treatment. Fertil Steril 98:. https://doi.org/10.1016/j.fertnstert.2012.08.011 10.1016/j.fertnstert.2012.08.011 22959459

[35] Alihocagil Emec Z, Ejder Apay S, Ozorhan EY (2017). Determination and comparison of sexual dysfunctions of women with and without infertility problems. Sex Disabil.

[36] Iris A, Aydogan Kirmizi D, Taner CE (2013). Effects of infertility and infertility duration on female sexual functions. Arch Gynecol Obstet.

[37] Zare Z, Golmakani N, Amirian M (2017). Comparison of sexual problems in fertile and infertile couples. J Caring Sci.

[38] Fallahzadeh H, Zareei Mahmood Abadi H, Momayyezi M (2019). The comparison of depression and anxiety between fertile and infertile couples: a meta-analysis study. Int J Reprod Biomed.

[39] Davari Tanha F, Mohseni M, Ghajarzadeh M (2014). Sexual function in women with primary and secondary infertility in comparison with controls. Int J Impot Res.

[40] Millheiser LS, Helmer AE, Quintero RB (2010). Is infertility a risk factor for female sexual dysfunction? A case-control study. Fertil Steril.

[41] Oskay UY, Beji NK, Serdaroglu H (2010). The issue of infertility and sexual function in Turkish women. Sex Disabil.

[42] Ozturk S, Sut HK, Kucuk L (2019). Examination of sexual functions and depressive symptoms among infertile and fertile women. Pakistan J Med Sci.

[43] Shahraki Z, Tanha FD, Ghajarzadeh M (2018) Depression, sexual dysfunction and sexual quality of life in women with infertility. BMC Womens Health 18:. https://doi.org/10.1186/s12905-018-0584-2 10.1186/s12905-018-0584-2 PMC6001164 29898709

[44] Diamond MP, Legro RS, Coutifaris C (2017). Sexual function in infertile women with polycystic ovary syndrome and unexplained infertility. Am J Obstet Gynecol.

[45] Czyżkowska A, Awruk K, Janowski K (2016). Sexual satisfaction and sexual reactivity in infertile women: the contribution of the dyadic functioning and clinical variables. Int J Fertil Steril.

[46] Hegyi BE, Kozinszky Z, Badó A (2019). Anxiety and depression symptoms in infertile men during their first infertility evaluation visit. J Psychosom Obstet Gynecol.

[47] Nyberg LM, Bennett AH, Burton BT (1993). Impotence. J Am Med Assoc.

[48] Berger DM (1980). Impotence following the discovery of azoospermia. Fertil Steril.

[49] Yikilmaz TN, Öztürk E, Hamidi N (2019). Evaluation of sexual dysfunction prevalence in infertile men with non-obstructive azoospermia. Arch Ital di Urol e Androl.

[50] O’Brien JH, Lazarou S, Deane L (2005). Erectile dysfunction and andropause symptoms in infertile men. J Urol.

[51] Gao J, Zhang X, Su P (2013). Relationship between sexual dysfunction and psychological burden in men with infertility: a large observational study in China. J Sex Med.

[52] Satkunasivam R, Ordon M, Hu B (2014). Hormone abnormalities are not related to the erectile dysfunction and decreased libido found in many men with infertility. Fertil Steril.

[53] Ozkan B, Orhan E, Aktas N, Coskuner ER (2015). Depression and sexual dysfunction in Turkish men diagnosed with infertility. Urology.

[54] Ma J, Zhang Y, Bao B, et al (2021) Prevalence and associated factors of erectile dysfunction, psychological disorders, and sexual performance in primary vs. secondary infertility men. Reprod Biol Endocrinol 19:. Doi: 10.1186/s12958-021-00720-5 10.1186/s12958-021-00720-5 PMC7942006 33750412

[55] Lotti F, Corona G, Rastrelli G (2012). Clinical correlates of erectile dysfunction and premature ejaculation in men with couple infertility. J Sex Med.

[56] Lotti F, Corona G, Castellini G (2016). Semen quality impairment is associated with sexual dysfunction according to its severity. Hum Reprod.

[57] Coward RM, Stetter C, Kunselman A (2019). Fertility related quality of life, gonadal function and erectile dysfunction in male partners of couples with unexplained infertility. J Urol.

[58] Song SH, Kim DS, Yoon TK (2016). Sexual function and stress level of male partners of infertile couples during the fertile period. BJU Int.

[59] Yang B, Xu P, Shi Y (2018). Erectile dysfunction and associated risk factors in Chinese males of infertile couples. J Sex Med.

[60] Elia J, Delfino M, Imbrogno N, Mazzilli F (2010). The impact of a diagnosis of couple subfertility on male sexual function. J Endocrinol Invest.

[61] Müller MJ, Schilling G, Haidl G (1999). Sexual satisfaction in male infertility. Arch Androl.

[62] Ramezanzadeh F, Aghssa MM, Jafarabadi M, Zayeri F (2006). Alterations of sexual desire and satisfaction in male partners of infertile couples. Fertil Steril.

[63] Lynch CD, Sundaram R, Maisog JM (2014). Preconception stress increases the risk of infertility: results from a couple-based prospective cohort study-the LIFE study. Hum Reprod.

[64] Shindel AW, Nelson CJ, Naughton CK, Mulhall JP (2008). Premature ejaculation in infertile couples: prevalence and correlates. J Sex Med.

[65] Kruljac M, Finnbogadóttir H, Bobjer J (2020). Symptoms of sexual dysfunction among men from infertile couples: prevalence and association with testosterone deficiency. Andrology.

[66] Colonnello E, Ciocca G, Limoncin E (2021). Redefining a sexual medicine paradigm: subclinical premature ejaculation as a new taxonomic entity. Nat Rev Urol.

[67] Sansone A, Aversa A, Corona G (2020). Management of premature ejaculation: a clinical guideline from the Italian society of andrology and sexual medicine (SIAMS). J Endocrinol Invest.

[68] Jannini EA, Nappi RE (2018). Couplepause: a new paradigm in treating sexual dysfunction during menopause and andropause. Sex Med Rev.

[69] Yeoh SH, Razali R, Sidi H, et al (2014) The relationship between sexual functioning among couples undergoing infertility treatment: A pair of perfect gloves. Compr Psychiatry 55:. https://doi.org/10.1016/j.comppsych.2012.09.002 10.1016/j.comppsych.2012.09.002 23116967

[70] Gabr AA, Omran EF, Abdallah AA (2017). Prevalence of sexual dysfunction in infertile versus fertile couples. Eur J Obstet Gynecol Reprod Biol.

[71] Shindel AW, Nelson CJ, Naughton CK (2008). Sexual function and quality of life in the male partner of infertile couples: prevalence and correlates of dysfunction. J Urol.

[72] Ciocca G, Rossi R, Collazzoni A (2020). The impact of attachment styles and defense mechanisms on psychological distress in a non-clinical young adult sample: a path analysis. J Affect Disord.

[73] Ciocca G, Zauri S, Limoncin E (2020). Attachment style, sexual orientation, and biological sex in their relationships with gender role. Sex Med.

[74] Mollaioli D, Sansone A, Ciocca G (2021). Benefits of sexual activity on psychological, relational, and sexual health during the COVID-19 breakout. J Sex Med.

[75] Purcell-Lévesque C, Brassard A, Carranza-Mamane B, Péloquin K (2018) Attachment and sexual functioning in women and men seeking fertility treatment. J Psychosom Obstet Gynecol 1–9. https://doi.org/10.1080/0167482X.2018.1471462 10.1080/0167482X.2018.1471462 29749292

[76] Ying LY, Wu LH, Loke AY (2015). Gender differences in experiences with and adjustments to infertility: a literature review. Int J Nurs Stud.

[77] Yangin H, Kukulu K, Gulşen S (2016). A survey on the correlation between sexual satisfaction and depressive symptoms during infertility. Health Care Women Int.

[78] Pozza A, Dèttore D, Coccia ME (2019). Depression and anxiety in pathways of medically assisted reproduction: the role of infertility stress dimensions. Clin Pract Epidemiol Ment Heal.

[79] Cocchiaro T, Meneghini C, Dal Lago A (2020). Assessment of sexual and emotional distress in infertile couple: validation of a new specific psychometric tool. J Endocrinol Invest.

[80] Jannini EA, Lombardo F, Salacone P (2004). Treatment of sexual dysfunctions secondary to male infertility with sildenafil citrate. Fertil Steril.

[81] Daniluk JC (1988). Infertility: intrapersonal and interpersonal impact. Fertil Steril.

[82] Benazon N, Wright J, Sabourin S (1992). Stress, sexual satisfaction, and marital adjustment in infertile couples. J Sex Marital Ther.

[83] Marci R, Graziano A, Piva I, et al (2012) Procreative sex in infertile couples: The decay of pleasure? Health Qual Life Outcomes 10:140. https://doi.org/10.1186/1477-7525-10-140 10.1186/1477-7525-10-140 PMC3543253 23176107

[84] Volgsten H, Skoog Svanberg A, Ekselius L (2008). Prevalence of psychiatric disorders in infertile women and men undergoing in vitro fertilization treatment. Hum Reprod.

[85] Woods NF, Olshansky E, Draye MA (1991). Infertility: women’s experiences. Health Care Women Int.

[86] Wischmann T, Schilling K, Toth B (2014). Sexuality, self-esteem and partnership quality in infertile women and men. Geburtshilfe Frauenheilkd.

[87] Ghavi F, Jamale S, Mosalanejad L, Mosallanezhad Z (2015). A study of couple burnout in infertile couples. Glob J Health Sci.

[88] Lee TY, Sun GH, Chao SC (2001). The effect of an infertility diagnosis on the distress, marital and sexual satisfaction between husbands and wives in Taiwan. Hum Reprod.

[89] Drosdzol A, Skrzypulec V (2009). Evaluation of marital and sexual interactions of polish infertile couples. J Sex Med.

[90] Khademi A, Alleyassin A, Amini M, Ghaemi M (2008). Evaluation of sexual dysfunction prevalence in infertile couples. J Sex Med.

[91] Barut MU, Çoksüer H, Sak S (2018). Evaluation of sexual function in women with hypogonadotropic hypogonadism using the female sexual function index (FSFI) and the beck depression inventory (BDI). Med Sci Monit.

[92] Freeman EW, Boxer AS, Rickels K (1985). Psychological evaluation and support in a program of in vitro fertilization and embryo transfer. Fertil Steril.

[93] Shoji M, Hamatani T, Ishikawa S, et al (2014) Sexual Satisfaction of infertile couples assessed using the Golombok-Rust Inventory of Sexual Satisfaction (GRISS). Sci Rep 4:. https://doi.org/10.1038/srep05203 10.1038/srep05203 PMC5381476 24902628

[94] Bayar U, Basaran M, Atasoy N (2014). Sexual dysfunction in infertile couples: evaluation and treatment of infertility. J Pak Med Assoc.

[95] Ohl J, Reder F, Fernandez A (2009). Impact de l’infertilité et de l’Assistance médicale à la procréation sur la sexualité. Gynecol Obstet Fertil.

[96] Bechoua S, Hamamah S, Scalici E (2016). Male infertility: an obstacle to sexuality?. Andrology.

[97] Coëffin-Driol C, Giami A (2004). The impact of infertility and its treatment on sexual life and marital relationships: review of the literature. Gynecol Obstet Fertil.

[98] Saleh RA, Ranga GM, Raina R (2003). Sexual dysfunction in men undergoing infertility evaluation: a cohort observational study. Fertil Steril.

[99] Jannini EA, Lenzi A, Isidori A, Fabbri A (2006). Subclinical erectile dysfunction: proposal for a novel taxonomic category in sexual medicine. J Sex Med.

[100] Cao HM, Wan Z, Gao Y (2019). Psychological burden prediction based on demographic variables among infertile men with sexual dysfunction. Asian J Androl.

[101] Bakhtiari A, Basirat Z, Nasiri-Amiri F (2016). Sexual dysfunction in women undergoing fertility treatment in Iran: prevalence and associated risk factors. J Reprod Infertil.

[102] Gungor ES, Seval O, Ilhan G, Verit FF (2018). Effect of intrauterine insemination treatment on sexual function and quality of life for infertile women. Pakistan J Med Sci.

[103] Smith NK, Madeira J, Millard HR (2015). Sexual function and fertility quality of life in women using in vitro fertilization. J Sex Med.

[104] Karli P, Ozdemir A (2019) Sexual dysfunction in in-vitro fertilization (IVF) patients and the effect of ovarian reserve on sexual dysfunction. Ann Med Res 1. https://doi.org/10.5455/annalsmedres.2019.06.350

[105] Facchin F, Somigliana E, Busnelli A (2019). Infertility-related distress and female sexual function during assisted reproduction. Hum Reprod.

[106] Lewis RW, Fugl-Meyer KS, Bosch R (2004). Epidemiology/risk factors of sexual dysfunction. J Sex Med.

[107] Maseroli E, Scavello I, Rastrelli G (2018). Outcome of medical and psychosexual interventions for vaginismus: a systematic review and meta-analysis. J Sex Med.

[108] De Souza M do CB, Gusmão MCG, Antunes RA, et al (2018) Vaginismus in assisted reproductive technology centers: An invisible population in need of care. J Bras Reprod Assist 22:35–41. Doi: 10.5935/1518-0557.20180013 10.5935/1518-0557.20180013 PMC5844657 29257632

[109] Watts G, Nettle D (2010). The role of anxiety in vaginismus: a case-control study. J Sex Med.

[110] Ramli M, Nora MZ, Roszaman R, Hatta S (2012). Vaginismus and subfertility: case reports on the association observed in clinical practice. Malaysian Fam Physician.

[111] Ciocca G, Limoncin E, Di Tommaso S (2013). Alexithymia and vaginismus: a preliminary correlation perspective. Int J Impot Res.

[112] Omani-Samani R, Amini P, Navid B (2019). Prevalence of sexual dysfunction among infertile women in Iran: a systematic review and meta-analysis. Int J Fertil Steril.

[113] Carter J, Applegarth L, Josephs L, et al (2011) A cross-sectional cohort study of infertile women awaiting oocyte donation: The emotional, sexual, and quality-of-life impact. Fertil Steril 95:. https://doi.org/10.1016/j.fertnstert.2010.10.004 10.1016/j.fertnstert.2010.10.004 21055740

[114] Pozza A, Dèttore D, Coccia ME (2020). Quality of life and infertility stress in homologous and heterologous medically assisted reproduction: the role of common and specific psychopathological traits. Perspect Psychiatr Care.

[115] de Mouzon J, Chambers GM, Zegers-Hochschild F (2020). International committee for monitoring assisted reproductive technologies world report: assisted reproductive technology 2012. Hum Reprod.

[116] Milazzo A, Mnatzaganian G, Elshaug AG (2016). Depression and anxiety outcomes associated with failed assisted reproductive technologies: a systematic review and meta-analysis. PLoS ONE.

[117] Brandes M, Van Der Steen JOM, Bokdam SB (2009). When and why do subfertile couples discontinue their fertility care? A longitudinal cohort study in a secondary care subfertility population. Hum Reprod.

[118] Van Eekelen R, Tjon-Kon-Fat RI, Bossuyt PMM (2018). Natural conception rates in couples with unexplained or mild male subfertility scheduled for fertility treatment: a secondary analysis of a randomized controlled trial. Hum Reprod.

[119] Sejbaek CS, Hageman I, Pinborg A (2013). Incidence of depression and influence of depression on the number of treatment cycles and births in a national cohort of 42 880 women treated with ART. Hum Reprod.

[120] Crawford NM, Hoff HS, Mersereau JE (2017). Infertile women who screen positive for depression are less likely to initiate fertility treatments. Hum Reprod.

[121] Stanhiser J, Steiner AZ (2018). Psychosocial aspects of fertility and assisted reproductive technology. Obstet Gynecol Clin North Am.

[122] Wincze JP (2015). Psychosocial aspects of ejaculatory dysfunction and male reproduction. Fertil Steril.

[123] Limoncin E, Lotti F, Rossi M (2016). The impact of premature ejaculation on the subjective perception of orgasmic intensity: validation and standardisation of the ‘Orgasmometer’. Andrology.

[124] Mollaioli D, Di Sante S, Limoncin E (2018). Validation of a visual analogue scale to measure the subjective perception of orgasmic intensity in females: the orgasmometer-F. PLoS ONE.

[125] Jannini EA, McCabe MP, Salonia A (2010). Organic vs. psychogenic? The Manichean diagnosis in sexual medicine. J Sex Med.

[126] Rosen RC (2001). Psychogenic erectile dysfunction: classification and management. Urol Clin North Am.

[127] Jannini EA, Droupy S (2019). Needs and expectations of patients with erectile dysfunction: an update on pharmacological innovations in phosphodiesterase type 5 inhibition with focus on sildenafil. Sex Med.

[128] Corona G, Rastrelli G, Burri A (2016). The safety and efficacy of Avanafil, a new 2nd generation PDE5i: comprehensive review and meta-analysis. Expert Opin Drug Saf.

[129] Pomara G, Morelli G, Canale D (2007). Alterations in sperm motility after acute oral administration of sildenafil or tadalafil in young, infertile men. Fertil Steril.

[130] Dong L, Zhang X, Yan X, et al (2021) Effect of Phosphodiesterase-5 inhibitors on the treatment of male infertility: a systematic review and meta-analysis. World J Mens Health 9:e11: doi: 10.5534/wjmh.200155 10.5534/wjmh.200155 PMC8443990 33663030

[131] McMahon CG, Jannini EA, Serefoglu EC, Hellstrom WJG (2016). The pathophysiology of acquired premature ejaculation. Transl Androl Urol.

[132] Gillman N, Gillman M (2019). Premature ejaculation: aetiology and treatment strategies. Med Sci.

[133] Jannini EA, Mcmahon C, Chen J (2011). The controversial role of phosphodiesterase type 5 inhibitors in the treatment of premature ejaculation. J Sex Med.

[134] Berger MH, Messore M, Pastuszak AW, Ramasamy R (2016). Association between infertility and sexual dysfunction in men and women. Sex Med Rev.

[135] Djaali W, Abdurrohim K, Helianthi DR (2019). Management of acupuncture as adjuvant therapy for in vitro fertilization. Med Acupunct.

